# Altered Frequencies and Functions of Innate Lymphoid Cells in Melanoma Patients Are Modulated by Immune Checkpoints Inhibitors

**DOI:** 10.3389/fimmu.2022.811131

**Published:** 2022-01-31

**Authors:** Costanza Maria Cristiani, Mariaelena Capone, Cinzia Garofalo, Gabriele Madonna, Domenico Mallardo, Marilena Tuffanelli, Vito Vanella, Marta Greco, Daniela Patrizia Foti, Giuseppe Viglietto, Paolo Antonio Ascierto, Hergen Spits, Ennio Carbone

**Affiliations:** ^1^ Department of Experimental and Clinical Medicine, “Magna Graecia” University of Catanzaro, Catanzaro, Italy; ^2^ Istituto Nazionale Tumori, IRCCS, Fondazione G. Pascale, Napoli, Italy; ^3^ Department of Health Sciences, “Magna Graecia” University of Catanzaro, Catanzaro, Italy; ^4^ Department of Experimental Immunology, University Medical Centres (UMC), University of Amsterdam, Amsterdam, Netherlands; ^5^ Department of Microbiology, Tumor and Cell Biology (MTC), Karolinska Institutet, Biomedicum, Stockholm, Sweden

**Keywords:** innate lymphoid cells, cytokines, melanoma, immune checkpoints inhibitors, nivolumab

## Abstract

Monoclonal antibodies targeting immune checkpoints improved clinical outcome of patients with malignant melanoma. However, the mechanisms are not fully elucidated. Since immune check-point receptors are also expressed by helper innate lymphoid cells (ILCs), we investigated the capability of immune checkpoints inhibitors to modulate ILCs in metastatic melanoma patients as well as melanoma cells effects on ILC functions. Here, we demonstrated that, compared to healthy donors, patients showed a higher frequency of total peripheral ILCs, lower percentages of CD117^+^ ILC2s and CD117^+^ ILCs as well as higher frequencies of CD117^-^ ILCs. Functionally, melanoma patients also displayed an impaired TNFα secretion by CD117^-^ ILCs and CD117^+^ ILCs. Nivolumab therapy reduced the frequency of total peripheral ILCs but increased the percentage of CD117^-^ ILC2s and enhanced the capability of ILC2s and CD117^+^ ILCs to secrete IL-13 and TNFα, respectively. Before Nivolumab therapy, high CCL2 serum levels were associated with longer Overall Survival and Progression Free Survival. After two months of treatment, CD117^-^ ILC2s frequency as well as serum concentrations of IL-6, CXCL8 and VEGF negatively correlated with both the parameters. Moreover, melanoma cells boosted TNFα production in all ILC subsets and increased the number of IL-13 producing ILC2s *in vitro*. Our work shows for the first time that PD-1 blockade is able to affect ILCs proportions and functions in melanoma patients and that a specific subpopulation is associated with the therapy response.

## Introduction

Innate lymphoid cells (ILCs) are a heterogeneous family of lymphocytes representing the innate counterpart of T cells and are involved in immune responses as well as in tissue development and homeostasis. Based on the cytokine secretion profiles and the expression of lineage-determining transcription factors, ILCs can be divided in five subsets: Natural Killer (NK) cells, ILC1s, ILC2s, ILC3s and Lymphoid Tissue inducer (LTi) cells. NK cells are involved in antiviral and antitumoral responses; they depend on Eomes for their development and secrete interferon γ (IFNγ) and tumor necrosis factor α (TNFα) (1). ILC1s show the same cytokine pattern but express only T-bet, responding to intracellular pathogens and mirroring T helper 1 (Th1) cells. ILC2s react against parasites producing Th2 cytokines such as interleukin (IL)-4, IL-5, IL-9, IL-13; their development depends on GATA3. ILC3s resemble Th17 cells, expressing RORγt and producing IL-17 and IL-22, and are involved in the immune response against extracellular microbes. NKp44 (NCR2) expression distinguishes two subsets of ILC3s, with NKp44^-^ ILC3s mainly producing IL-17, while IL-22 secretion is restricted to NKp44^+^ ILC3s. LTi cells are mainly found in the embryo, where they drive the development of secondary lymphoid tissues. Similar to ILC3s, they rely on RORγt but do not express NCRs (2). Recent work has made it clear that in peripheral blood CD117^+^ ILCs from healthy individuals, which are mostly comprising ILC3s in tissues, are a mix of precursors that can develop into various ILC subsets and more mature ILC3s. CD117^-^ ILCs in peripheral blood include ILC1s but are probably heterogeneous as well (3, 4). CD117 also distinguishes ILC2s in two subsets: CD117^-^ ILC2s are considered to be a more mature and lineage-committed subpopulation, expressing high levels of GATA3 and secreting large amounts of type 2 cytokines; on the other hand, CD117^+^ ILC2s are more plastic and share some features with ILC3s, including RORγt expression and capability to produce IL-17 (5, 6).

ILCs are mainly tissue-resident cells, where they constantly scan the microenvironment. Thus, their localization and function suggest that ILCs may be the first immune cells to sense solid tumor cells. In addition, ILCs in peripheral blood potentially detect circulating metastatic tumor cells (7). However, whether and how ILCs are involved in tumor immunity depends on the specific subset(s) involved, the cytokines they produce, as well as the tumor microenvironment (TME) they are in. Since ILCs are highly plastic cells, the TME may also change function and phenotype of ILC subsets (2).

Human malignant melanoma cells are highly metastatic and resistant to conventional therapies. Until recent years, prognosis of metastatic melanoma patients was poor, with a 5-year overall survival rate lower than 10%. However, immunotherapy with immune checkpoint inhibitors (ICIs) dramatically changed the clinical outcome. The three monoclonal antibodies currently used for metastatic melanoma immunotherapy are Ipilimumab, targeting Cytotoxic T-Lymphocyte Antigen (CTLA)-4, and Nivolumab and Pembrolizumab, inhibiting Programmed Cell Death Protein (PD)-1. Ipilimumab improved patients’ survival to 20% (8), a rate that has been further raised by PD-1 blocking and combined therapies (9, 10). However, it is still unclear why only a fraction of patients responds to ICIs. Currently, response evaluation is based mainly on clinical parameters, while cellular and molecular variables have been less extensively investigated.

T cells represent the main target of ICIs; however, checkpoint receptors are also expressed by ILCs. PD-1 displays the broadest expression pattern, being found on all the mature ILC subsets as well as on progenitors (11–13). On the other side, CTLA-4 expression has been observed only in mature ILCs but not in precursors (11). Thus, it is possible that immune checkpoints blockade can modify the functions of ILCs.

In this work, we analyzed the oscillations in peripheral ILC subsets in metastatic melanoma patients during ICI therapies. In addition, we investigated the interactions of ILCs with melanoma cells.

## Materials and Methods

### Study Design

For the characterization of ILC compartment during melanoma immunotherapy, a total of 32 stage IV melanoma patients were enrolled at the Istituto Nazionale Tumori Fondazione “G. Pascale” of Naples, Italy, granted ethical permission. Of them, 8 patients were treated with Ipilimumab (anti CTLA-4), while the remaining were treated with Nivolumab (anti PD-1). For both the cohorts, patients were naïve or previously underwent to different types of therapies, including chemotherapies, targeted therapies and immunotherapies.

Written informed consent was obtained from all patients in accordance with the Declaration of Helsinki for the use of human biological samples for research purposes. For each patient, blood samples were collected before the starting and after two months of treatment. Clinical characteristics of enrolled patients are summarized in **Supplementary Table 1**.

Thirty-three sex- and age-matched healthy donors were enrolled at the Pugliese-Ciaccio Hospital and University Magna Graecia of Catanzaro, Catanzaro, Italy. Experiments were performed once per sample.

### Isolation of Peripheral Blood Mononuclear Cells and Innate Lymphoid Cells

Blood samples from melanoma patients were collected in 6 mL EDTA vacutainer tubes (BD diagnostics), while healthy donors samples derived from buffycoats. Peripheral blood mononuclear cells (PBMCs) were isolated by density gradient centrifugation (Ficoll-Paque, GE Healthcare) within 2h of sample collection and frozen in 90% FBS + 10% DMSO freezing medium.

For functional experiments, buffycoats were provided by the blood bank (Sanquin, Amsterdam). ILCs were isolated from healthy donors as previously described (14). Briefly, fresh PBMCs were enriched for ILCs by negative selection using immunomagnetic cell sorting. Negative selection was performed by using CD3, CD14, CD16, and CD19 biotin-conjugated antibodies with the Mojosort magnetic cell separation system (BioLegend). Cell suspensions were then stained with antibodies against lineage (CD1a, CD3, CD4, CD5, CD11c, CD14, CD16, CD19, CD34, CD123, BDCA2, TCRαβ, TCRγδ and FcER1α), CD3, CD127, CD117 and CRTH2 to be sorted by 3-laser FACSAria. The three main peripheral ILC subsets were sorted: CD127^+^CRTH2^-^CD117^-^ ILCs (CD117^-^ ILCs), CD127^+^CRTH2^+^CD117^+/-^ ILCs (ILC2s) and CD127^+^CRTH2^-^CD117^+^ ILCs (CD117^+^ ILCs). Detailed information about used antibodies can be found in **Supplementary Table 2**.

### Cell Cultures

Based on informed consent, melanoma cell lines were obtained from surgical specimens. CNF cell line derived from a primary tumor of a patient admitted at the Fondazione IRCCS Istituto Nazionale dei Tumori, Milan (2009). AMM16 cell line derived from a lymph node metastasis of a patient admitted at the San Raffaele University Hospital, Milan (2013). Both the melanoma cell lines were maintained in RPMI-1640 medium (Life Technology) supplemented with penicillin (100 IU/mL) and streptomycin (100 mg/mL) and 10% FBS and used within 3 weeks after thawing.

For co-culture experiments, the three main peripheral ILC subpopulations (CD117^-^ ILCs, ILC2s and CD117^+^ ILCs) were freshly *ex vivo*-isolated by sorting and co-cultured in allogenic setting with melanoma cells at an E:T ratio of 5:1, in 96-well round-bottom plates for 72 hours at 37°C. Yssel’s medium [IMDM supplemented with 4% (vol/vol) Yssel’s supplement (made in-house; AMC) and 1% [vol/vol] human AB serum (Invitrogen)] supplemented with IL-2 (20 U/ml; Novartis) and IL-7 (20 ng/mL; Pepro Tech) was used. For each experimental point, 5-10 x 10^4^ ILCs were used and melanoma cells were scaled accordingly. During the last three hours of co-culture, cells were stimulated with phorbol 12-myristate 13-acetate (PMA) (10 ng/ml; Sigma) plus Ionomycin (500 nM; Merck) in the presence of Golgi Plug (BD Biosciences). The same activation protocol was used to measure cytokine productions from patients and healthy donors ILCs.

### Flow Cytometry Analysis

Thawed PBMCs, as well as fresh ILCs, were subjected to immunofluorescence staining. Cells were washed in PBS 1X and stained with the LIVE/DEAD Fixable Blue Dead Cell Stain Kit (eBioscience) for 30 minutes at 4°C, followed by surface antigens staining with antibodies (**Supplementary Table 2**) in PBS 1X again for 30 minutes at 4°C. For cytokines intracellular staining, PMA/ionomycin-activated cells were fixed and permeabilized using the Foxp3/Transcription Factor Staining Buffer Kit (Thermo Fisher Scientific) for 20 minutes at 4°C and intracellular cytokines were stained with antibodies for 30 min at 4°C in permeabilization buffer. Cells were then washed and resuspended in PBS 1X, acquired on LSRFortessa (BD Bioscience) and analyzed with FlowJo software version 10 (Treestar US).

### Cytokine Assays

Quantification of serum cytokines and growth factors was performed on thawed samples deriving from the supernatants obtained after peripheral blood centrifugation. Samples from 24 melanoma patients and 15 healthy donors were analyzed. The simultaneous quantification of the following cytokines: IL-2, IL-4, IL-6, IL-10, IL-1α, IL-1β, vascular endothelial growth factor (VEGF), C-C motif ligand 2 (CCL2), C-X-C motif ligand 8 (CXCL8), IFNγ, TNFα and epidermal growth factor (EGF) was performed by using the biochip analyzer Evidence Investigator and the chemiluminescent immunoassay “Cytokine Array I” kit (Randox Labs) (15). Concentrations of IL-13, IL-22, IL-23 (R&D Systems); IL-5, IL-17a (Diaclone SAS); tumor growth factor (TGF) β (DRG Instruments GmbH) were evaluated by ELISA kits. All the assays were performed following the manufacturer’s recommended procedure. For IL-13, IL-22, IL-23 and TGF-β, physiological ranges were provided by kits. For IL-5 and IL-17a, as well as cytokines quantified by the “Cytokine Array I”, physiological ranges were calculated by using values obtained from healthy donors.

### Statistical Analysis

Data obtained from multiple experiments were analyzed for statistical significance using the GraphPad Prism 5.0 software. Paired Student t-test or Wilcoxon signed-rank test were used to compare data from two related group. Data from unrelated groups were analyzed using unpaired Student t-test or Mann-Whitney U test. Normally distributed groups of data were analyzed by paired or unpaired Student’s t-test, while Wilcoxon signed-rank test and Mann-Whitney U test were used for not-normally distributed groups of data. Kaplan-Meier (KM) curves were used to compare Overall Survival (OS) and Progression-Free Survival (PFS) of patients. Both frequencies of ILC subpopulations and concentrations of serum cytokines were used as variables to generate KM curves and the median value of each variable, calculated on the entire cohort, was used as cut-off. KM curves comparison and p-value calculation was performed by log-rank test.

Overall, 53 variables were analyzed and 16 were found to be significantly different between samples groups. Based on the Bonferroni’s correction for multiple comparisons, reference p-value should be lowered to 0.00094 (0.05/53) in order to exclude variables resulting as significant by chance. However, under the null hypothesis of no association, less than 3 p-values (5% out of 53) would have expected to be <0.05. Instead, 30% (16 out 53) of p-values were found to be <0.05, which suggests the presence of actual associations (16). For this reason, p-value < 0.05 was kept as threshold for statistical significance in all the analyses.

## Results

### ICI Therapies Induce ILC Subsets Fluctuations in Stage IV Melanoma Patients

**Figure 1:**
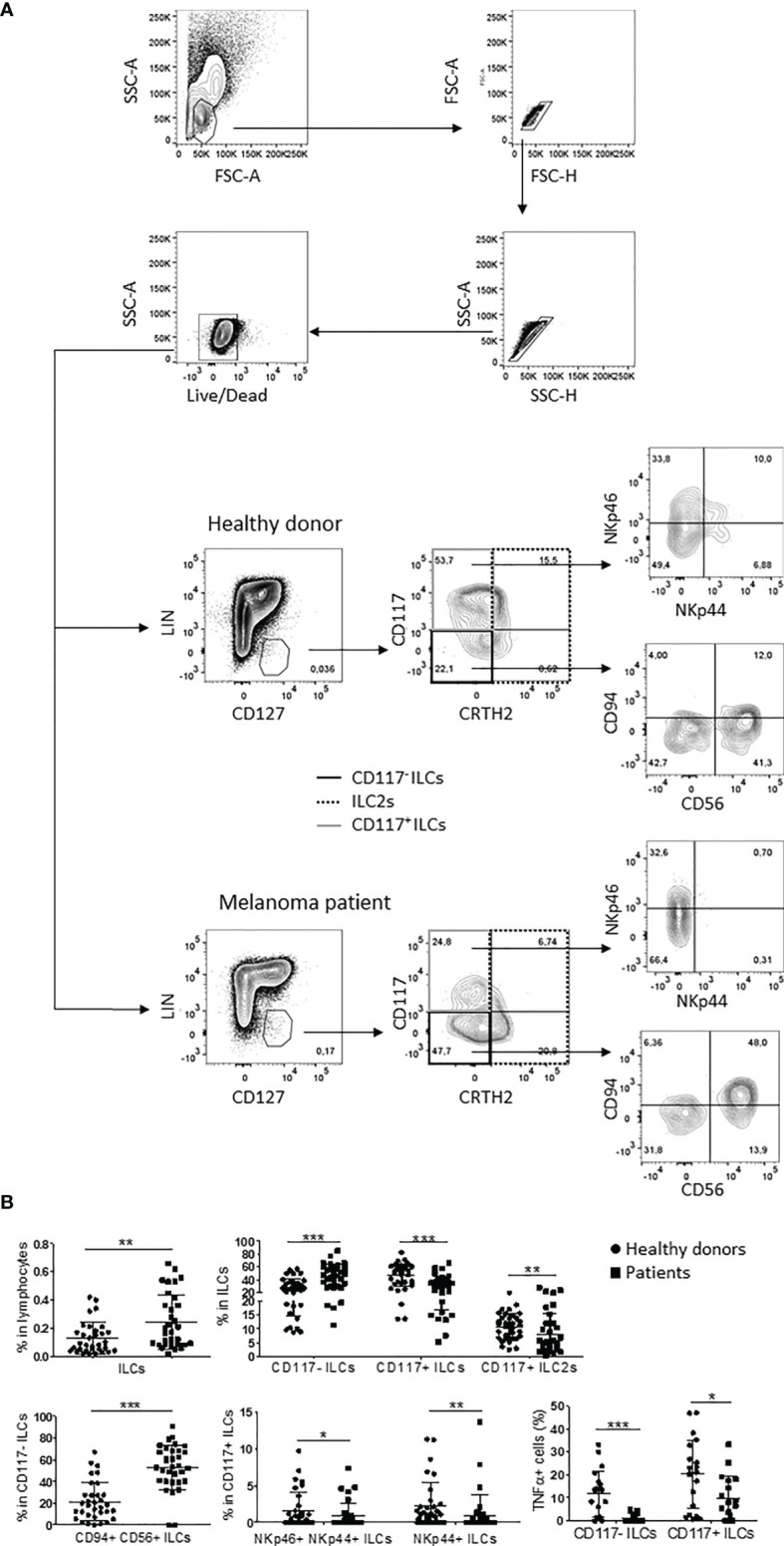
ILC subpopulations profile in melanoma patients and healthy donors. **(A)** Gating strategy and representative plots of peripheral ILC subsets frequencies in healthy donors and stage IV melanoma patients. **(B)** Statistical analysis of the indicated ILC subsets observed in 33 healthy donors (circles) and 32 stage IV melanoma patients (squares). For TNFα statistics, cumulative data from 20 healthy donors and 18 stage IV melanoma patients were used. The assay was performed once per sample. Analyses were performed by unpaired Student’s t-test for normally distributed samples or Mann-Whitney test for not-normally distributed samples. Cumulative data are shown as mean ± SD. ***p-value < 0.001; **p-value < 0.01; *p-value < 0.05.

To investigate the frequencies of blood ILC subsets during metastatic melanoma disease, we performed a phenotypical analysis on a cohort of 32 stage IV melanoma patients and 33 healthy donors using a 14-colors multiparametric cytofluorimetric approach. The frequency of peripheral ILCs was higher in melanoma patients, as previously demonstrated in hematopoietic tumors (17, 18). Furthermore, the three main ILC subsets exhibited altered proportions compared to healthy donors, with lower percentages of CD117^+^ ILC2s and CD117^+^ ILCs, which are mostly composed of precursor committed to ILC3s and also include more mature ILC3s (3, 4), and higher frequencies of CD117^-^ ILCs, consistent with previous observations in melanoma (19). Of notice, among CD117^-^ ILCs, CD94^+^CD56^+^ ILC1-like cells were expanded, as was reported previously in leukemia patients (20), while the frequencies of NKp46^+^NKp44^+^ and NKp44^+^ mature ILC3s within CD117^+^ ILCs were reduced (**Figures 1A, B**).

Moreover, CD117^-^ ILCs and CD117^+^ ILCs from melanoma patients showed impaired TNFα secretion when activated *ex vivo* with PMA/ionomycin in comparison with ILCs from healthy individuals (**Figure 1B**), consistent with observations in hematological malignancies (17, 18).

**Figure 2:**
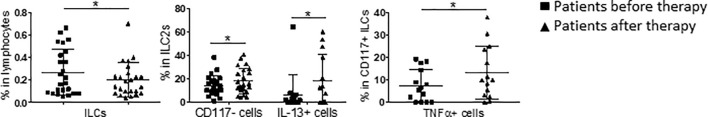
Oscillations in ILC subpopulations induced by Nivolumab treatment in stage IV melanoma patients. Statistical analysis of the frequency of the indicated ILC subsets in stage IV melanoma patients before (squares) and after (triangles) two months of Nivolumab therapy. For total ILCs and CD117^-^ ILC2s, the analysis was performed cumulating data from 24 patients. For IL-13 and TNFα, the analysis was performed cumulating data from 14 patients. Analyses were performed by paired Student’s t-test for normally distributed samples or Wilcoxon matched-pairs signed rank test for not-normally distributed samples. Cumulative data are shown as mean ± SD. *p-value < 0.05.

To investigate the modifications in ILC subset frequencies that could occur in stage IV melanoma patients during ICI therapies, we analyzed patients treated with Ipilimumab (8 patients) or Nivolumab (24 patients) separately. For both cohorts, the patients were sampled before the first cycle of therapy and near to the first clinical assessment (about at two months). While Ipilimumab did not appear to significantly affect ILC subsets (data not shown), Nivolumab did. Particularly, anti-PD-1 ICI induced a reduction in the frequency of the total peripheral ILCs while it increased at the same time the percentage of mature CD117^-^ ILC2s (**Figure 2**). Furthermore, anti PD-1 treatment enhanced IL-13 and TNFα secretion by ILC2s and CD117^+^ ILCs, respectively (**Figure 2**). IL-13 up-regulation by ILC2s was mainly due to the CD117^+^ subset, while the secretory activity of CD117^-^ ILCs was not affected by Nivolumab (**Supplementary Figure 1**). On the other side, although PD-1 was detectable on all the investigated ILC subsets, neither its frequency nor its levels of expression were shown to be affected by metastatic melanoma disease or Nivolumab treatment (**Supplementary Figure 2**).

Overall, these data indicate that the frequencies of ILCs in the peripheral blood of melanoma patients are changed and that Nivolumab therapy is able to affect ILC subsets, both modulating their frequencies and secretory activity. This effect is particularly apparent on ILC2s.

### Serum Cytokines in Nivolumab-Treated Melanoma Patients

To monitor serum cytokine profiles and their potential alternations induced by treatment, we analyzed serum samples from 24 stage IV melanoma patients before and after Nivolumab therapy. We focused on cytokines produced by ILCs and/or affecting their biology. Though Nivolumab did not significantly alter serum cytokine profiles, in melanoma patients we observed a deviation from the physiological range for 7 out 18 cytokines tested: IL-1β, IL-6, CCL2, CXCL8, VEGF, IL-5 and IL-13 (**Table 1**). While the latter two are mainly produced during parasitic infections and allergic reactions, the others are known be pro-inflammatory and/or angiogenetic factors (21). Particularly, serum levels of IL-6, CXCL8 and CCL2 have been previously shown to increase during melanoma progression (22). Interestingly, although the difference did not reach the statistical significance, the levels of IL-1β and CCL2 were similar before and after treatment, while IL-6, CXCL8, VEGF and IL-13 concentrations tended to further increase and IL-5 levels tended to decrease after Nivolumab treatment. However, detectable levels of IL-5 and IL-13 could be found only in 5 and 7 out of tested samples, respectively (data not shown).

**Table 1:** Serum cytokines/chemokines deviating from physiological range in Nivolumab-treated stage IV melanoma patients.

Cytokine	Physiological range (pg/ml)	Mean ± SD before treatment (pg/ml)	Mean ± SD after treatment (pg/ml)	p-value
IL-1β	0.2 – 2.4	3.28 ± 1.57	3.69 ± 2.88	0.86
IL-6	0.2 – 5.6	9.57 ± 11.22	20.56 ± 30.11	0.36
CCL2	70.5 – 209.3	468.1 ± 202.8	474.9 ± 217.3	0.50
CXCL8	1.9 – 17.4	56.05 ± 78.97	138.40 ± 88.61	0.70
VEGF	15.5 – 431.0	385.0 ± 256.0	461.1 ± 338.9	0.51
IL-5	0.0 – 5.0	14.52 ± 54.54	6.07 ± 22.2	0.25
IL-13	Undetectable	2.73 ± 6.82	10.91 ± 17.79	0.18

The analysis was performed by paired Student’s t-test for normally distributed samples or Wilcoxon matched-pairs signed rank test for not-normally distributed samples cumulating data from 24 patients.

### ILC Subsets and Serum Cytokines Correlate With Survival in Nivolumab-Treated Melanoma Patients

Next, we investigated the association between single ILC variables, serum cytokines profiles and therapy outcome in Nivolumab-treated patients. KM curves were generated by using the median value of each considered variable to determine outcome by months of OS and PFS. Comparison between KM curve and p-value calculations were performed by log-rank test.

**Figure 3:**
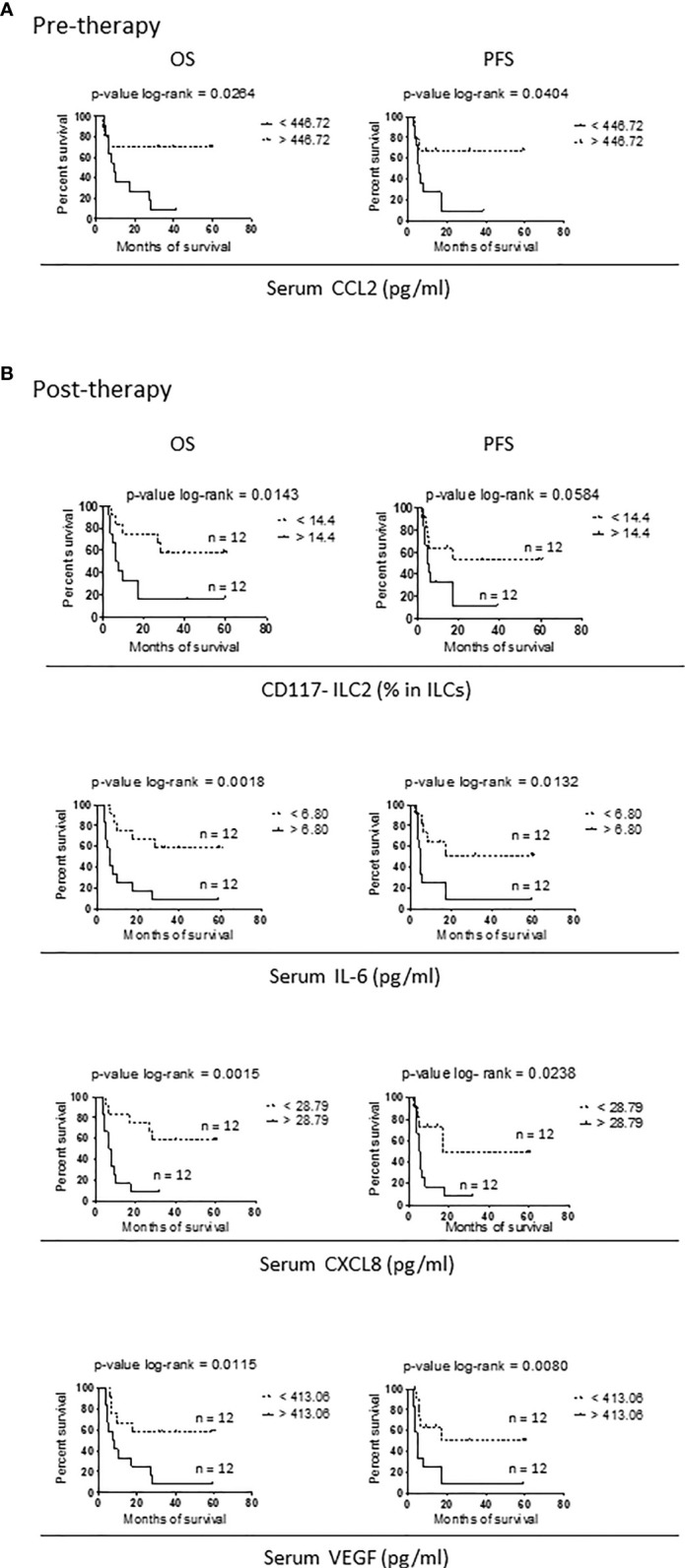
Variables correlating with survival in Nivolumab-treated stage IV melanoma patients. **(A, B)** Kaplan-Meier curves for OS (right panels) and PFS (left panels) in short survival (solid lines) and long survival (dotted lines) stage IV melanoma patients before therapy **(A)** and after two months of Nivolumab treatment **(B)**. Data from 24 patients were used. For each variable, the median value calculated on the entire cohort was chosen as cut-off and reported on the right of the related panels. Log-rank test was used to compare curves. p-values are reported on the top of each panel.

At baseline, CCL2 only was associated with the response to Nivolumab therapy, in that higher serum concentrations of CCL2 were associated with both longer OS and PFS (**Figure 3A**).

When the association with survival was addressed after two months of therapy, four parameters were identified: the frequency of mature CD117^-^ ILC2s and serum levels of IL-6, CXCL8 and VEGF.

For all the variables, patients with values below the cut-off showed a longer survival. Again, the association was significant or close to significance for both OS and PFS (**Figure 3B**).

Thus, in melanoma patients in which Nivolumab improves OS and PFS, there are reduced serum levels of pro-inflammatory and pro-angiogenic cytokines and a low percentage of peripheral mature CD117^-^ ILC2. This suggests that Nivolumab could reduce inflammation and modulate circulating ILC2s in responding patients.

### TNFα and IL-13 Production by ILCs Is Up-Regulated by Melanoma Cells *In Vitro*

Finally, we addressed the capability of CD117^-^ ILCs, ILC2s and CD117^+^ ILCs to respond to melanoma cells in co-culture experiments by measuring their cytokine production by flow cytometry. Thus, circulating ILCs sorted from healthy donors were co-cultured for three days with two different primary melanoma cell lines at 5:1 ratio, and then stimulated with PMA/ionomycin for the last three hours of co-culture. Primary melanoma cells phenotype was previously characterized and is summarized in **Supplementary Figure 3** (22).

**Figure 4:**
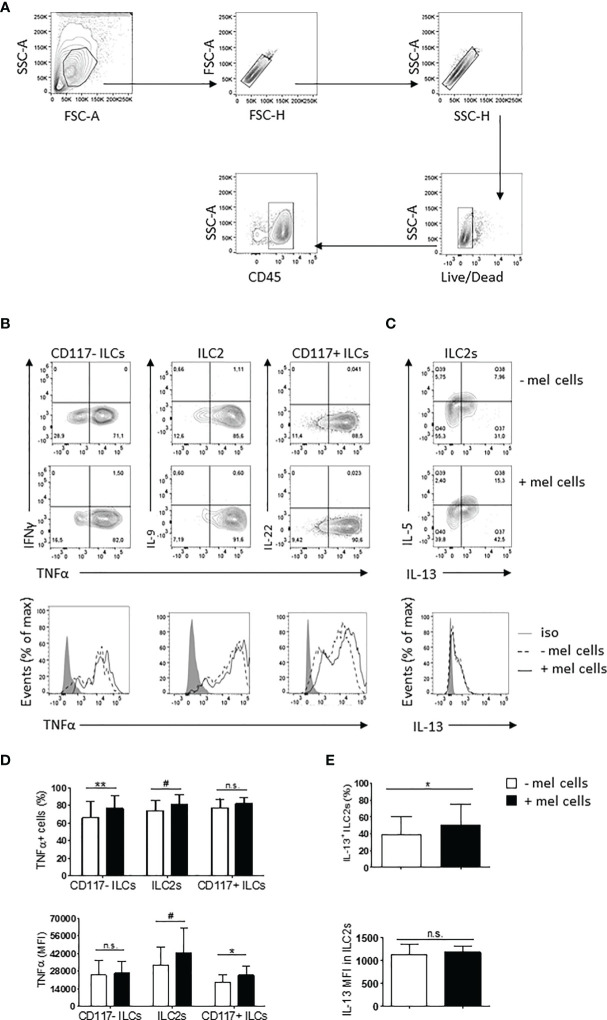
Cross-talk between ILC subsets and melanoma cells *in vitro*. **(A)** Gating strategy used to identify ILCs within co-cultures. **(B, C)** Representative plots of TNFα up-regulation in all the three ILC subsets **(B)** and IL-13 up-regulation in ILC2s **(C)** co-cultured with melanoma cells at 5:1 ratio for 3 days. **(D, E)** Percentage of positive cells (upper lines) and expression (lower lines) of TNFα in all the three ILC subsets **(D)** and of IL-13 in ILC2s **(E)**. Cumulative data derived from eight independent experiments performed using two different melanoma cell lines are shown as mean ± SD. For each cell line, at least three independent experiments were carried out. Analysis was performed by paired Student t test. **p-value < 0.01; *p-value < 0.05; ^#^p-value < 0.1. n.s. stand for Not Significant.

Identification of ILCs within co-culture was performed as shown in **Figure 4A**. Melanoma cells addition up-regulated TNFα in all the three ILC subsets and IL-13 in ILC2s (**Figures 4B–E** and **Supplementary Figure 4**). Particularly, the frequency of TNFα^+^CD117^-^ ILCs (**Figures 4B, D**), the level of expression of TNFα in CD117^+^ ILCs (**Figures 4B, D**) and the frequency of IL-13^+^ ILC2s (**Figures 4C, E**) showed a significant increase, while both the expression and the frequency of TNFα^+^ positive cells in ILC2s tended to be higher although the difference was not statistically significant (**Figures 4B, D**). Notably, the activation of the CD117^-^ ILCs with PMA/ionomycin did not induce IFNγ production, ruling out the possibility of contamination with NK cells (**Figure 4B**).

Taken together, these data suggest a cross-talk between melanoma cells and ILCs occurring *in vitro*. The net result of this cross-talk *in vivo* on melanoma pathophysiology might depend on the microenvironment and the relative contribution of each subset.

## Discussion

Because of their localization within tissues and their capability to promptly react to activating stimuli, ILCs may be involved in an early stage of an immune response to cancer cells. While the role played by NK cells in melanoma is firmly established (23, 24), our knowledge about the potential role of other ILC subpopulations is limited.

In this work, we showed that metastatic melanoma, similarly to haematological malignancies (17, 18), induced the expansion of peripheral ILCs. We observed changes in the relative proportions of ILC subsets, with a reduction of CD117^-^ ILCs and a parallel increasing of CD117^+^ ILCs frequency, a pattern also observed in colorectal carcinoma (25). In a recent paper, Ercolano et al. also observed CD117^-^ ILCs expansion, but not CD117^+^ ILCs contraction, nor total ILCs increase (19). The discrepancy with this study might be due to the differences in the patient cohorts, since in that study stage III patients were also enrolled while we focused exclusively on stage IV patients.

Particularly, we observed an increase in the frequency of the CD94^+^CD56^+^ ILC1-like subpopulation within CD117^-^ ILCs and a reduction in the percentage of NKp46^+^NKp44^+^ and NKp44^+^ mature ILC3s within CD117^+^ ILCs. Mature NKp44^+^ ILC3s have been proposed to have a positive role against melanoma. In mouse models, locally produced IL-12 induced their accumulation, which in turn increased adhesion molecules expression on tumor vasculature and leukocyte infiltration (26, 27). On the other hand, the role of ILC1s in melanoma is more controversial. Because of their capability to polarize a Th1 response, ILC1s may protect against tumors (7). Specifically, the CD94^+^CD56^+^ ILC1-like subpopulation is thought to possess some attributes of NK cells and cytotoxic activity (20, 28). However, in the melanoma TME, TGFβ converts NK cells into non-cytotoxic ILC1-like cells, characterized by an immune suppressive phenotype and a metastasis-promoting activity mediated by TNFα (29, 30).

Of notice, we demonstrated that peripheral ILC frequencies are affected not only by metastatic melanoma but also by the immune therapy with Nivolumab. Particularly, we showed for the first time that Nivolumab induced a contraction in total blood ILCs with a concomitant expansion of mature CD117^-^ ILC2s Additionally, we showed that lower peripheral frequencies of mature CD117^-^ ILC2 were positively associated with survival benefit. To the best of our knowledge, it is the first time in which a specific peripheral ILC subpopulation has been associated to clinical outcome in solid tumors. Although such correlation needs to be confirmed in a larger cohort, it suggests that mature CD117^-^ ILC2s might play an important role in response to Nivolumab. Indeed, ILC2s have been shown to promote anti-melanoma immune surveillance by the induction of eosinophilia in mouse models (31, 32). Such effect was further boosted by PD-1 blockade, which has been also shown to promote ILC2s proliferation (32). Thus, the expansion of CD117^-^ ILC2s might be a phenomenon induced by Nivolumab regardless of the clinical outcome, but the survival benefit would depend on the effective capability of this subset to migrate from the periphery to the tumor in order to exert its anti-melanoma activity. Indeed, ILC2s enrichment within the tumor has been associated to higher survival in patients with pancreatic ductal adenocarcinoma (33).

At functional level, we showed an impaired secretion of TNFα by both CD117^-^ ILCs and CD117^+^ ILCs from melanoma patients, as reported in blood tumors (17, 18), and the capability of Nivolumab to enhance the secretion of IL-13 and TNFα by ILC2s and CD117^+^ ILCs, respectively. The latter is in line with previous works showing that PD-1 engagement reduces the secretory activity in ILC2s and CD117^+^ ILCs, including ILC3s, but not CD117^-^ ILCs (34–36). Interestingly, the cytokines whose secretion was improved by Nivolumab were the same that were found to be up-regulated by melanoma cells in co-culture experiments. Indeed, we observed an increased secretion of TNFα by all the three subsets and of IL-13 specifically by ILC2s. Although usually considered a member of type 2 immunity, ILC2s have been shown to be able to produce TNFα in response to different stimuli (6, 37). Particularly, Maggi et al. demonstrated that activation with PMA/iono could induce TNFα secretion by ILC2s (37). The reduced secretory capability of ILCs from melanoma patients has been demonstrated also by Ercolano et al. (19), although these authors showed that the impairment was mainly limited to IFNγ. Additionally, they demonstrated that melanoma cells were able to reduce IFNγ secretion by this subset while TNFα was not affected (19), which is in contrast with the TNFα up-regulation we observed. However, since co-culture settings differ in the two studies, a direct comparison of the results is challenging.

Both TNFα and IL-13 have been suggested to exert a positive role against melanoma cells. TNFα exerts indirect cytotoxic effects against melanoma by inducing the destruction of tumor vasculature (38). Indeed, TNFα is successfully used to treat *in transit* melanoma metastases and its presence within tumors has been shown to be induced by and synergize with ICIs in melanoma patients (39, 40), while its systemic blockage is associated with a reduced survival (41). On the other hand, IL-13 can recruit and activate neutrophils and macrophages within the tumor lesion, with cytotoxic activity against melanoma cells (42, 43).

Our *in vitro* data indicate that the direct interaction between ILCs and melanoma cells results in the up-regulation of cytokines secretion by the former. A reasonable hypothesis explaining such an activation is the engagement of NCRs expressed on ILCs (44) by the cognate ligands expressed on melanoma cells (**Supplementary Figure 2**). By contrast, although PD-1 is expressed on ILCs (**Supplementary Figure 1**) and melanoma cells express PD-L1 (**Supplementary Figure 2**), their interaction would not be sufficient to inhibit cytokines secretion by ILCs. Thus, ILCs activation would be mediated by activating signals delivered by NCRs and not counteracted by PD-1-mediated inhibition. In turn, this suggests that Nivolumab might improve the function of ILC2s and CD117^+^ ILCs not by directly affecting the interaction between melanoma cells and ILCs but rather by disrupting additional interactions mediated by PD-1 *in vivo*. Melanoma TME is enriched with suppressive immune cell subsets expressing PD-L1, such as macrophages, myeloid-derived suppressive cells (MDSCs) and regulatory T cells (Tregs) (24, 45). ILC2s activity has been shown to be limited by suppressive subsets through cell-to-cell contact (46–49), while similar data on CD117^+^ ILCs are missing. Although a defined role for PD-L1-mediated inhibition toward ILC2s has been demonstrated only for macrophages (46), it is likely that also MDSCs and Tregs might exploit PD-1/PD-L1 axis to suppress ILCs function. However, a direct inhibition of ILCs mediated by melanoma cells *in vivo* through PD-L1 is still possible, since PD-L1 expression has been shown to be up-regulated by several pro-tumoral mediators found within TME (45).

**Figure 5:**
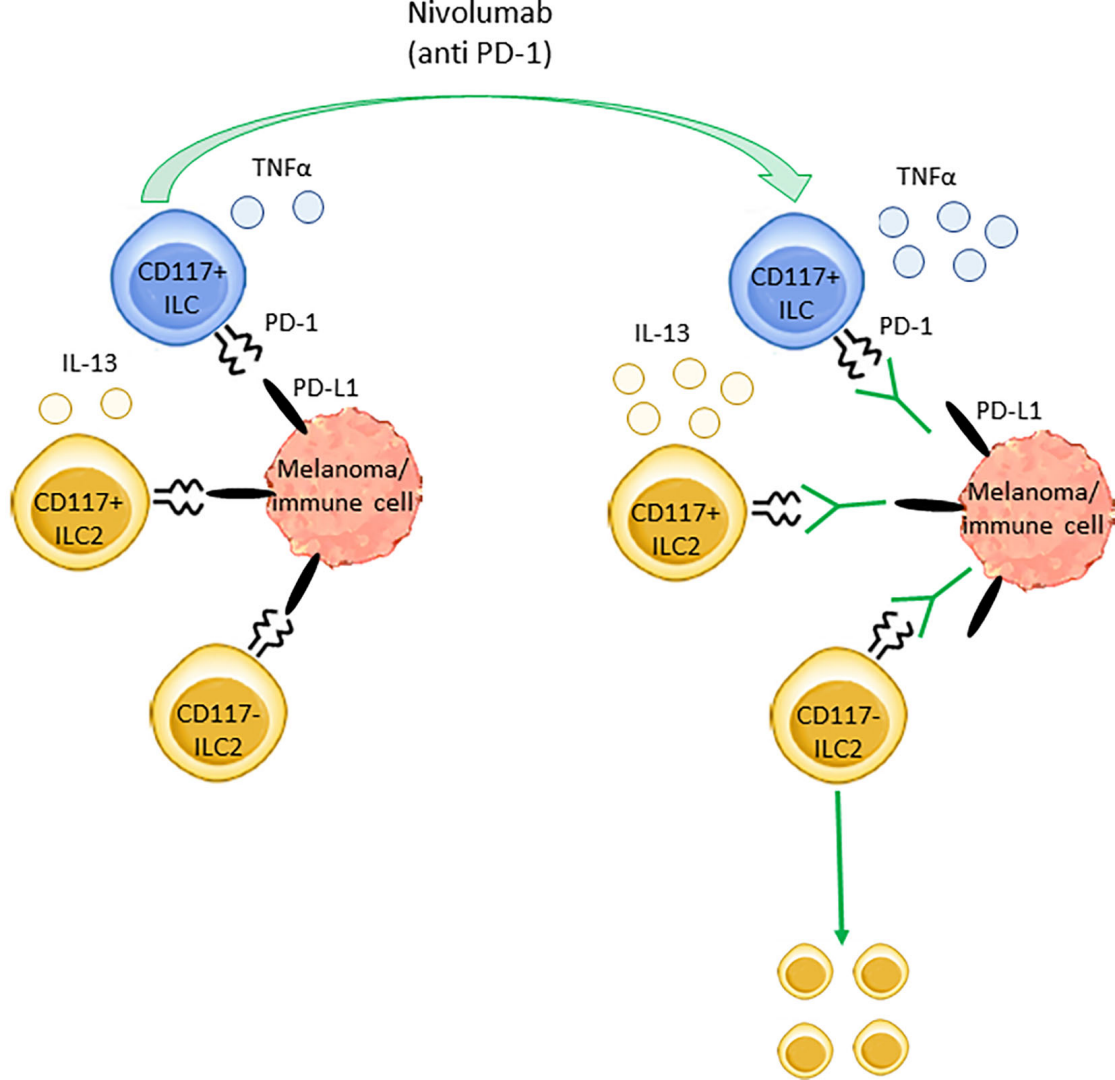
Nivolumab effects on ILCs subsets in stage IV melanoma. The inhibitory receptor PD-1 regulates the secretory activity of both ILC2s and ILC3s as well as CD117^+^ ILC precursors. In stage IV melanoma patients, PD-1 targeting through Nivolumab enhances the production of TNFα and IL-13 from circulating CD117^+^ ILCs and CD117^+^ ILC2s, respectively. Furthermore, Nivolumab induces the expansion of blood CD117^-^ ILC2s. These effects are likely due to Nivolumab-mediated disruption of the interaction between PD-1, expressed on ILCs, and its ligand PD-L1, expressed on melanoma cells and/or other immune cells such as macrophages, MDSCs and Tregs.

Collectively, data suggest that Nivolumab can act not only on T cells but also on specific ILC subsets restoring their proliferation and/or cytokines production by interfering with the inhibitory interactions between ILCs and suppressive immune cell subsets and/or melanoma cells (**Figure 5**).

In contrast to what has been recently described by Rethacker et al. (50), we did not observe any change in ILC frequencies induced by Ipilimumab. This could depend on the numerical size of the cohort as well as the different gating strategy.

Finally, we reported that high blood levels of CCL2 and low concentrations of IL-6, CXCL8 and VEGF correlated with better OS and PFS. Specifically, for CCL2 such association was found before the start of the therapy, while for the other cytokines it could be observed after two months of treatment. IL-6, CXCL8 and VEGF have been widely demonstrated to be related to worse clinical outcome in melanoma. They are well-known autocrine factors promoting melanoma invasiveness (51–53), which suggests that their high concentrations would associate with a more aggressive tumor phenotype and a reduced survival. Indeed, high serum levels of CXCL8 and IL-6 in melanoma have been associated with melanoma progression (22), higher tumor burden (54–57), and shorten PFS and OS in patients treated with biochemotherapy (54–56), Ipilimumab (57–59) and Nivolumab (58, 59). Similar associations for VEGF have also been reported (54, 60, 61). To our knowledge, it is the first time that a correlation between VEGF and survival has been demonstrated in melanoma patients treated with Nivolumab (61). However, this observation should be confirmed in larger cohorts.

On the other hand, serum CCL2 has been poorly investigated as potential biomarker in melanoma patients. A recent study reported that high systemic concentrations of CCL2 were positively associated with OS in melanoma patients with *in transit* metastasis treated with melphalan (62), possibly because of the capability of this chemokine to attract both T lymphocytes and monocytes within melanoma (63).

Aside from the prototypical cytokines, ILCs can secrete pro-inflammatory cytokines after specific stimuli. Particularly, both ILC2s and CD117^+^ ILCs have been shown to be able to express CXCL8 (64, 65), while IL-6 and VEGF production is restricted to ILC2s (66, 67). However, we did not find any association between serum cytokines concentrations and peripheral ILC frequencies in Nivolumab-treated patients (data not shown), consistently with the scarce presence of these cells in the blood. On the other hand, ILC2s have been demonstrated to express CCR2 (68, 69), the cognate receptor for CCL2, which may represent a potential mechanism recruiting ILC2s within melanoma.

Thus, the low levels of serum IL-6, CXCL8 and VEGF observed in Nivolumab-responding patients after two months of therapy might reflect, in these subjects, the effective capability of the treatment to counteract chronic inflammation-driven progression of the tumor, while the high concentrations of CCL2 might indicate a better infiltration of immune cells within neoplastic lesions, both resulting in longer OS and PFS.

Overall, we showed here for the first time that Nivolumab treatment, targeting PD-1, is able to affect frequencies and functions of specific ILC subsets and selectively activate them in metastatic melanoma patients. Of notice, we preliminary demonstrated that mature CD117^-^ ILC2s are associated with survival. Additionally, we reported that activated ILCs are able to secrete cytokines in response to melanoma cells. Collectively, these data suggest that ILCs might represent an important and until now poorly appreciated immune cell subset playing a role in mediating the anti-tumoral response induced by Nivolumab in metastatic melanoma patients.

## Data Availability Statement

The raw data supporting the conclusions of this article will be made available by the authors, without undue reservation.

## Ethics Statement

The studies involving human participants were reviewed and approved by the institutional review board of Istituto Nazionale Tumori IRCCS Fondazione “G. Pascale” (Naples, Italy) (protocol code 33/17oss, January 10, 2018). The patients/participants provided their written informed consent to participate in this study.

## Author Contributions

EC, HS, and PA conceived and designed the study. CC, MC, CG, GM, DM, MT and MG performed the experiments. CC, VV, DF, GV, HS and EC analyzed and interpreted the data. CC drafted the work. All the authors critically read and revised the manuscript and approved the submitted version.

## Funding

This work was supported by Italian Association for Cancer Research (IG15521 and IG20312); Wenner-Gren Foundation, Sweden; Fondazione Melanoma Onlus, Naples; Ministero dell’Istruzione, dell’Università e della Ricerca (MIUR) PRIN 2017 2017M8YMR8_002; Fondazione NIBIT, Siena, Italy (grants to EC) and Italian Ministry of Health (IT-MOH) through “Ricerca Corrente”, grants number M2-2 (grants to PAA). CMC was supported by EFIS-IL Short Term Fellowship and EACR Travel Fellowship.

## Conflict of Interest

PA has/had a consultant/advisory role for Bristol Myers Squibb, Roche-Genentech, Merck Sharp & Dohme, Novartis, Array, Merck Serono, Pierre-Fabre, Incyte, Medimmune, AstraZeneca, Syndax, Sun Pharma, Sanofi, Idera, Ultimovacs, Sandoz, Immunocore, 4SC, Alkermes, Italfarmaco, Nektar, Boehringer-Ingelheim, Eisai, Regeneron, Daiichi Sankyo, Pfizer, Oncosec, Nouscom, Takis, Lunaphore. He also received research funding from Bristol Myers Squibb, Roche-Genentech, Array, Sanofi and travel support from MSD. HS has a consultant role for SGK. All outside the submitted work.

The remaining authors declare that the research was conducted in the absence of any commercial or financial relationships that could be construed as a potential conflict of interest.

## Publisher’s Note

All claims expressed in this article are solely those of the authors and do not necessarily represent those of their affiliated organizations, or those of the publisher, the editors and the reviewers. Any product that may be evaluated in this article, or claim that may be made by its manufacturer, is not guaranteed or endorsed by the publisher.

## References

[1] Cristiani CM Palella E Sottile R Tallerico R Garofalo C Carbone E. Human NK Cell Subsets in Pregnancy and Disease: Toward a New Biological Complexity. Front Immunol (2016) 7:656. doi: 10.3389/fimmu.2016.00656 28082990 PMC5187385

[2] Vivier E Artis D Colonna M Diefenbach A Di Santo JP Eberl G. Innate Lymphoid Cells: 10 Years on. Cell (2018) 174(5):1054–66. doi: 10.1016/j.cell.2018.07.017 30142344

[3] Lim AI Verrier T Vosshenrich CA Di Santo JP. Developmental Options and Functional Plasticity of Innate Lymphoid Cells. Curr Opin Immunol (2017) 44:61–8. doi: 10.1016/j.coi.2017.03.010 28359987

[4] Nagasawa M Heesters BA Kradolfer CMA Krabbendam L Martinez-Gonzalez I de Bruijn MJW. KLRG1 and NKp46 Discriminate Subpopulations of Human CD117^+^CRTH2- ILCs Biased Toward ILC2 or ILC3. J Exp Med (2019) 216(8):1762–76. doi: 10.1084/jem.20190490 PMC6683990 31201208

[5] Bal SM Bernink JH Nagasawa M Groot J Shikhagaie MM Golebski K. IL-1β, IL-4 and IL-12 Control the Fate of Group 2 Innate Lymphoid Cells in Human Airway Inflammation in the Lungs. Nat Immunol (2016) 17(6):636–45. doi: 10.1038/ni.3444 27111145

[6] Golebski K Ros XR Nagasawa M van Tol S Heesters BA Aglmous H. IL-1β, IL-23, and TGF-β Drive Plasticity of Human ILC2s Towards IL-17-Producing ILCs in Nasal Inflammation. Nat Commun (2019) 10(1):2162. doi: 10.1038/s41467-019-09883-7 31089134 PMC6517442

[7] Artis D Spits H. The Biology of Innate Lymphoid Cells. Nature (2015) 517(7534):293–301. doi: 10.1038/nature14189 25592534

[8] Hodi FS O’Day SJ McDermott DF Weber RW Sosman JA Haanen JB. Improved Survival With Ipilimumab in Patients With Metastatic Melanoma. N Engl J Med (2010) 363(8):711–23. doi: 10.1056/NEJMoa1003466 PMC3549297 20525992

[9] Robert C Long GV Brady B Dutriaux C Maio M Mortier L. Nivolumab in Previously Untreated Melanoma Without BRAF Mutation. N Engl J Med (2015) 372(4):320–30. doi: 10.1056/NEJMoa1412082 25399552

[10] Larkin J Chiarion-Sileni V Gonzalez R Grob JJ Rutkowski P Lao CD. Five-Year Survival With Combined Nivolumab and Ipilimumab in Advanced Melanoma. N Engl J Med (2019) 381(16):1535–46. doi: 10.1056/NEJMoa1910836 31562797

[11] Salimi M Wang R Yao X Li X Wang X Hu Y. Activated Innate Lymphoid Cell Populations Accumulate in Human Tumour Tissues. BMC Cancer (2018) 18(1):341. doi: 10.1186/s12885-018-4262-4 29587679 PMC5870240

[12] Yu Y Tsang JC Wang C Clare S Wang J Chen X. Single-Cell RNA-Seq Identifies a PD-1(Hi) ILC Progenitor and Defines Its Development Pathway. Nature (2016) 539(7627):102–6. doi: 10.1038/nature20105 27749818

[13] Seillet C Mielke LA Amann-Zalcenstein DB Su S Gao J Almeida FF. Deciphering the Innate Lymphoid Cell Transcriptional Program. Cell Rep (2016) 17(2):436–47. doi: 10.1016/j.celrep.2016.09.025 27705792

[14] Krabbendam L Nagasawa M Spits H Bal SM. Isolation of Human Innate Lymphoid Cells. Curr Protoc Immunol (2018) 122(1):e55. doi: 10.1002/cpim.55 29957859

[15] Accattato F Greco M Pullano SA Carè I Fiorillo AS Pujia A. Effects of Acute Physical Exercise on Oxidative Stress and Inflammatory Status in Young, Sedentary Obese Subjects. PLoS One (2017) 12(6):e0178900. doi: 10.1371/journal.pone.0178900 28582461 PMC5459463

[16] Sottile R Tannazi M Johansson MH Cristiani CM Calabró L Ventura V. NK- and T-Cell Subsets in Malignant Mesothelioma Patients: Baseline Pattern and Changes in the Context of Anti-CTLA-4 Therapy. Int J Cancer (2019) 145(8):2238–48. doi: 10.1002/ijc.32363 31018250

[17] Trabanelli S Curti A Lecciso M Salomé B Riether C Ochsenbein A. CD127^+^ Innate Lymphoid Cells Are Dysregulated in Treatment Naïve Acute Myeloid Leukemia Patients at Diagnosis. Haematologica (2015) 100(7):257–60. doi: 10.3324/haematol.2014.119602 PMC4486236 25710455

[18] de Weerdt I van Hoeven V Munneke JM Endstra S Hofland T Hazenberg MD. Innate Lymphoid Cells Are Expanded and Functionally Altered in Chronic Lymphocytic Leukemia. Haematologica (2016) 101(11):e461–4. doi: 10.3324/haematol.2016.144725 PMC5394863 27662009

[19] Ercolano G Garcia-Garijo A Salomé B Gomez-Cadena A Vanoni G Mastelic-Gavillet B. Immunosuppressive Mediators Impair Proinflammatory Innate Lymphoid Cell Function in Human Malignant Melanoma. Cancer Immunol Res (2020) 8(4):556–64. doi: 10.1158/2326-6066.CIR-19-0504 32019778

[20] Salomé B Gomez-Cadena A Loyon R Suffiotti M Salvestrini V Wyss T. CD56 as a Marker of an ILC1-Like Population With NK Cell Properties That Is Functionally Impaired in AML. Blood Adv (2019) 3(22):3674–87. doi: 10.1182/bloodadvances.2018030478 PMC6880898 31765481

[21] Altan-Bonnet G Mukherjee R. Cytokine-Mediated Communication: A Quantitative Appraisal of Immune Complexity. Nat Rev Immunol (2019) 19(4):205–17. doi: 10.1038/s41577-019-0131-x PMC8126146 30770905

[22] Cristiani CM Turdo A Ventura V Apuzzo T Capone M Madonna G. Accumulation of Circulating CCR7(+) Natural Killer Cells Marks Melanoma Evolution and Reveals a CCL19-Dependent Metastatic Pathway. Cancer Immunol Res (2019) 7(5):841–52. doi: 10.1158/2326-6066.CIR-18-0651 30940644

[23] Cristiani CM Garofalo C Passacatini LC Carbone E. New Avenues for Melanoma Immunotherapy: Natural Killer Cells? Scand J Immunol (2020) 91(4):e12861. doi: 10.1111/sji.12861 31879979

[24] Garofalo C De Marco C Cristiani CM. NK Cells in the Tumor Microenvironment as New Potential Players Mediating Chemotherapy Effects in Metastatic Melanoma. Front Oncol (2021) 11:754541. doi: 10.3389/fonc.2021.754541 34712615 PMC8547654

[25] Loyon R Jary M Salomé B Gomez-Cadena A Galaine J Kroemer M. Peripheral Innate Lymphoid Cells Are Increased in First Line Metastatic Colorectal Carcinoma Patients: A Negative Correlation With Th1 Immune Responses. Front Immunol (2019) 10:2121. doi: 10.3389/fimmu.2019.02121 31555301 PMC6742701

[26] Eisenring M vom Berg J Kristiansen G Saller E Becheret B. IL-12 Initiates Tumor Rejection *via* Lymphoid Tissue-Inducer Cells Bearing the Natural Cytotoxicity Receptor Nkp46. Nat Immunol (2010) 11(11):1030–8. doi: 10.1038/ni.1947 20935648

[27] Nussbaum K Burkhard SH Ohs I Mair F Klose CSN Arnold SJ. Et. Tissue Microenvironment Dictates the Fate and Tumor-Suppressive Function of Type 3 ILCs. J Exp Med (2017) 214(8):2331–47. doi: 10.1084/jem.20162031 PMC5551572 28698286

[28] Krabbendam L Bernink JH Spits H. Innate Lymphoid Cells: From Helper to Killer. Curr Opin Immunol (2021) 68:28–33. doi: 10.1016/j.coi.2020.08.007 32971468

[29] Gao Y Souza-Fonseca-Guimaraes F Bald T Ng SS Young A Ngiow SF. Tumor Immunoevasion by the Conversion of Effector NK Cells Into Type 1 Innate Lymphoid Cells. Nat Immunol (2017) 18(9):1004–15. doi: 10.1038/ni.3800 28759001

[30] Cortez VS Ulland TK Cervantes-Barragan L Bando JK Robinette ML Wang Q. SMAD4 Impedes the Conversion of NK Cells Into ILC1-Like Cells by Curtailing Non-Canonical TGF-β Signaling. Nat Immunol (2017) 18(9):995–1003. doi: 10.1038/ni.3809 28759002 PMC5712491

[31] Ikutani M Yanagibashi T Ogasawara M Tsuneyama K Yamamoto S Hattori Y. Identification of Innate IL-5-Producing Cells and Their Role in Lung Eosinophil Regulation and Antitumor Immunity. J Immunol (2012) 188(2):703–13. doi: 10.4049/jimmunol.1101270 22174445

[32] Jacquelot N Seillet C Wang M Pizzolla A Liao Y Hediyeh-Zadeh S. Blockade of the Co-Inhibitory Molecule PD-1 Unleashes ILC2-Dependent Antitumor Immunity in Melanoma. Nat Immunol (2021) 22(7):851–64. doi: 10.1038/s41590-021-00943-z PMC7611091 34099918

[33] Moral JA Leung J Rojas LA Ruan J Zhao J Sethna Z. ILC2s Amplify PD-1 Blockade by Activating Tissue-Specific Cancer Immunity. Nature (2020) 579(7797):130–5. doi: 10.1038/s41586-020-2015-4 PMC7060130 32076273

[34] Taylor S Huang Y Mallett G Stathopoulou C Felizardo TC Sun MA. PD-1 Regulates KLRG1^+^ Group 2 Innate Lymphoid Cells. J Exp Med (2017) 214(6):1663–78. doi: 10.1084/jem.20161653 PMC5461001 28490441

[35] Vacca P Pesce S Greppi M Fulcheri E Munari E Olive D. PD-1 Is Expressed by and Regulates Human Group 3 Innate Lymphoid Cells in Human Decidua. Mucosal Immunol (2019) 12(3):624–31. doi: 10.1038/s41385-019-0141-9 30755717

[36] Tumino N Martini S Munari E Scordamaglia F Besi F Mariotti FR. Presence of Innate Lymphoid Cells in Pleural Effusions of Primary and Metastatic Tumors: Functional Analysis and Expression of PD-1 Receptor. Int J Cancer (2019) 145(6):1660–8. doi: 10.1002/ijc.32262 PMC6767381 30856277

[37] Maggi L Montaini G Mazzoni A Rossettini B Capone M Rossi MC. Human Circulating Group 2 Innate Lymphoid Cells Can Express CD154 and Promote IgE Production. J Allergy Clin Immunol (2017) 139(3):964–76.e4. doi: 10.1016/j.jaci.2016.06.032 27576126

[38] Balkwill F. Tumour Necrosis Factor and Cancer. Nat Rev Cancer (2009) 9(5):361–71. doi: 10.1038/nrc2628 19343034

[39] Deroose JP Grünhagen DJ van Geel AN de Wilt JH Eggermont AM Verhoef C. Long-Term Outcome of Isolated Limb Perfusion With Tumour Necrosis Factor-α for Patients With Melanoma in-Transit Metastases. Br J Surg (2011) 98(11):1573–80. doi: 10.1002/bjs.7621 21739427

[40] Vredevoogd DW Kuilman T Ligtenberg MA Boshuizen J Stecker KE de Bruijn B. Augmenting Immunotherapy Impact by Lowering Tumor TNF Cytotoxicity Threshold. Cell (2020) 180(2):404–5. doi: 10.1016/j.cell.2020.01.005 31978349

[41] Verheijden RJ May AM Blank CU Aarts MJB van den Berkmortel FWPJ van den Eertwegh AJM. Association of Anti-TNF With Decreased Survival in Steroid Refractory Ipilimumab and Anti-PD1-Treated Patients in the Dutch Melanoma Treatment Registry. Clin Cancer Res (2020) 26(9):2268–74. doi: 10.1158/1078-0432.CCR-19-3322 31988197

[42] Ma HL Whitters MJ Jacobson BA Donaldson DD Collins M Dunussi-Joannopoulos K. Tumor Cells Secreting IL-13 But Not IL-13Ralpha2 Fusion Protein Have Reduced Tumorigenicity *In Vivo* . Int Immunol (2004) 16(7):1009–17. doi: 10.1093/intimm/dxh105 15184346

[43] Ellyard JI Quah BJC Simson L Parish CR. Alternatively Activated Macrophage Possess Antitumor Cytotoxicity That Is Induced by IL-4 and Mediated by Arginase-1. J Immunother (2010) 33(5):443–52. doi: 10.1097/CJI.0b013e3181cd8746 20463604

[44] Guia S Fenis A Vivier E Narni-Mancinelli E. Activating and Inhibitory Receptors Expressed on Innate Lymphoid Cells. Semin Immunopathol (2018) 40(4):331–41. doi: 10.1007/s00281-018-0685-x 29789862

[45] Antonangeli F Natalini A Garassino MC Sica A Santoni A Di Rosa F. Regulation of PD-L1 Expression by NF-κb in Cancer. Front Immunol (2020) 11:584626. doi: 10.3389/fimmu.2020.584626 33324403 PMC7724774

[46] Oldenhove G Boucquey E Taquin A Acolty V Bonetti L Ryffel B. PD-1 Is Involved in the Dysregulation of Type 2 Innate Lymphoid Cells in a Murine Model of Obesity. Cell Rep (2018) 25(8):2053–60.e4. doi: 10.1016/j.celrep.2018.10.091 30463004

[47] Cao Y He Y Wang X Liu Y Shi K Zheng Z. Polymorphonuclear Myeloid-Derived Suppressor Cells Attenuate Allergic Airway Inflammation by Negatively Regulating Group 2 Innate Lymphoid Cells. Immunology (2019) 156(4):402–12. doi: 10.1111/imm.13040 PMC6418421 30575026

[48] Liu W Zeng Q Wen Y Tang Y Yan S Li Y. Inhibited Interleukin 35 Expression and Interleukin 35-Induced Regulatory T Cells Promote Type II Innate Lymphoid Cell Response in Allergic Rhinitis. Ann Allergy Asthma Immunol (2021) 126(2):152–61.e1. doi: 10.1016/j.anai.2020.08.005 32771356

[49] Rigas D Lewis G Aron JL Wang B Banie H Sankaranarayanan I. Type 2 Innate Lymphoid Cell Suppression by Regulatory T Cells Attenuates Airway Hyperreactivity and Requires Inducible T-Cell Costimulator-Inducible T-Cell Costimulator Ligand Interaction. J Allergy Clin Immunol (2017) 139(5):1468–77.e2. doi: 10.1016/j.jaci.2016.08.034 27717665 PMC5378695

[50] Rethacker L Roelens M Bejar C Maubec E Moins-Teisserenc H Caignard A. Specific Patterns of Blood ILCs in Metastatic Melanoma Patients and Their Modulations in Response to Immunotherapy. Cancers (Basel) (2021) 13(6):1446. doi: 10.3390/cancers13061446 33810032 PMC8004602

[51] Uen WC Hsieh CH Tseng TT Jiang SS Tseng JC Lee SC. Anchorage Independency Promoted Tumor Malignancy of Melanoma Cells Under Reattachment Through Elevated Interleukin-8 and CXC Chemokine Receptor 1 Expression. Melanoma Res (2015) 25(1):35–46. doi: 10.1097/CMR.0000000000000134 25426644

[52] Mohapatra P Prasad CP Andersson T. Combination Therapy Targeting the Elevated Interleukin-6 Level Reduces Invasive Migration of BRAF Inhibitor-Resistant Melanoma Cells. Mol Oncol (2019) 13(2):480–94. doi: 10.1002/1878-0261.12433 PMC6360505 30582770

[53] Lacal PM Ruffini F Pagani E D’Atri S. An Autocrine Loop Directed by the Vascular Endothelial Growth Factor Promotes Invasiveness of Human Melanoma Cells. Int J Oncol (2005) 27(6):1625–32. 16273219

[54] Ugurel S Rappl G Tilgen W Reinhold U. Increased Serum Concentration of Angiogenic Factors in Malignant Melanoma Patients Correlates With Tumor Progression and Survival. J Clin Oncol (2001) 19(2):577–83. doi: 10.1200/JCO.2001.19.2.577 11208853

[55] Mouawad R Rixe O Meric JB Khayat D Soubrane C. Serum Interleukin-6 Concentrations as Predictive Factor of Time to Progression in Metastatic Malignant Melanoma Patients Treated by Biochemotherapy: A Retrospective Study. Cytokines Cell Mol Ther (2002) 7(4):151–6. doi: 10.1080/13684730210002328 14660055

[56] Soubrane C Rixe O Meric JB Khayat D Mouawad R. Pretreatment Serum Interleukin-6 Concentration as a Prognostic Factor of Overall Survival in Metastatic Malignant Melanoma Patients Treated With Biochemotherapy: A Retrospective Study. Melanoma Res (2005) 15(3):199–204. doi: 10.1097/00008390-200506000-00009 15917702

[57] Sanmamed MF Carranza-Rua O Alfaro C Oñate C Martín-Algarra S Perez G. Serum Interleukin-8 Reflects Tumor Burden and Treatment Response Across Malignancies of Multiple Tissue Origins. Clin Cancer Res (2014) 20(22):5697–707. doi: 10.1158/1078-0432.CCR-13-3203 25224278

[58] Schalper KA Carleton M Zhou M Chen T Feng Y Huang SP. Elevated Serum Interleukin-8 Is Associated With Enhanced Intratumor Neutrophils and Reduced Clinical Benefit of Immune-Checkpoint Inhibitors. Nat Med (2020) 26(5):688–92. doi: 10.1038/s41591-020-0856-x PMC8127102 32405062

[59] Laino AS Woods D Vassallo M Qian X Tang H Wind-Rotolo M. Serum Interleukin-6 and C-Reactive Protein Are Associated With Survival in Melanoma Patients Receiving Immune Checkpoint Inhibition. J Immunother Cancer (2020) 8(1):e000842. doi: 10.1136/jitc-2020-000842 32581042 PMC7312339

[60] Wang X Montoyo-Pujol YG Bermudez S Corpas G Martin A Almazan F. Serum Cytokine Profiles of Melanoma Patients and Their Association With Tumor Progression and Metastasis. J Oncol (2021) 2021:6610769. doi: 10.1155/2021/6610769 33574842 PMC7861916

[61] Khattak MA Abed A Reid AL McEvoy AC Millward M Ziman M. Role of Serum Vascular Endothelial Growth Factor (VEGF) as a Potential Biomarker of Response to Immune Checkpoint Inhibitor Therapy in Advanced Melanoma: Results of a Pilot Study. Front Oncol (2020) 10:1041:1041. doi: 10.3389/fonc.2020.01041 32695680 PMC7338663

[62] Johansson J Kiffin R Aydin E Nilsson MS Hellstrand K Lindnér P. Isolated Limb Perfusion With Melphalan Activates Interferon-Stimulated Genes to Induce Tumor Regression in Patients With Melanoma in-Transit Metastasis. Oncoimmunology (2019) 9(1):1684126. doi: 10.1080/2162402X.2019.1684126 32002296 PMC6959433

[63] Nakasone Y Fujimoto M Matsushita T Hamaguchi Y Huu DL Yanaba M. Host-Derived MCP-1 and MIP-1α Regulate Protective Anti-Tumor Immunity to Localized and Metastatic B16 Melanoma. Am J Pathol (2012) 180(1):365–74. doi: 10.1016/j.ajpath.2011.09.005 22037251

[64] Xue L Salimi M Panse I Mjösberg JM McKenzie AN Spits H. Prostaglandin D2 Activates Group 2 Innate Lymphoid Cells Through Chemoattractant Receptor-Homologous Molecule Expressed on TH2 Cells. J Allergy Clin Immunol (2014) 133(4):1184–94. doi: 10.1016/j.jaci.2013.10.056 PMC3979107 24388011

[65] Carrega P Loiacono F Di Carlo E Scaramuccia A Mora M Conte R. NCR(+)ILC3 Concentrate in Human Lung Cancer and Associate With Intratumoral Lymphoid Structures. Nat Commun (2015) 6:8280. doi: 10.1038/ncomms9280 26395069

[66] Hardman CS Chen YL Salimi M Nahler J Corridoni D Jagielowicz M. IL-6 Effector Function of Group 2 Innate Lymphoid Cells (ILC2) Is NOD2 Dependent. Sci Immunol (2021) 6(59):eabe5084. doi: 10.1126/sciimmunol.abe5084 34021026 PMC7611333

[67] Shen X Pasha MA Hidde K Khan A Liang M Guan W. Group 2 Innate Lymphoid Cells Promote Airway Hyperresponsiveness Through Production of VEGFA. J Allergy Clin Immunol (2018) 141(5):1929–31.e4. doi: 10.1016/j.jaci.2018.01.005 29382598 PMC6019125

[68] Beuraud C Lombardi V Luce S Horiot S Naline E Neukirch C. CCR10^+^ ILC2s With ILC1-Like Properties Exhibit a Protective Function in Severe Allergic Asthma. Allergy (2019) 74(5):933–43. doi: 10.1111/all.13679 30475388

[69] Weston CA Rana BMJ Cousins DJ. Differential Expression of Functional Chemokine Receptors on Human Blood and Lung Group 2 Innate Lymphoid Cells. J Allergy Clin Immunol (2019) 143(1):410–3.e9. doi: 10.1016/j.jaci.2018.08.030 30205185 PMC6320261

