# Supplementary figures and images for: Altered Frequencies and Functions of Innate Lymphoid Cells in Melanoma Patients Are Modulated by Immune Checkpoints Inhibitors
Source: Front Immunol. 2022 Jan 31;13:811131. doi: 10.3389/fimmu.2022.811131 (PMC8841353; doi:10.3389/fimmu.2022.811131)

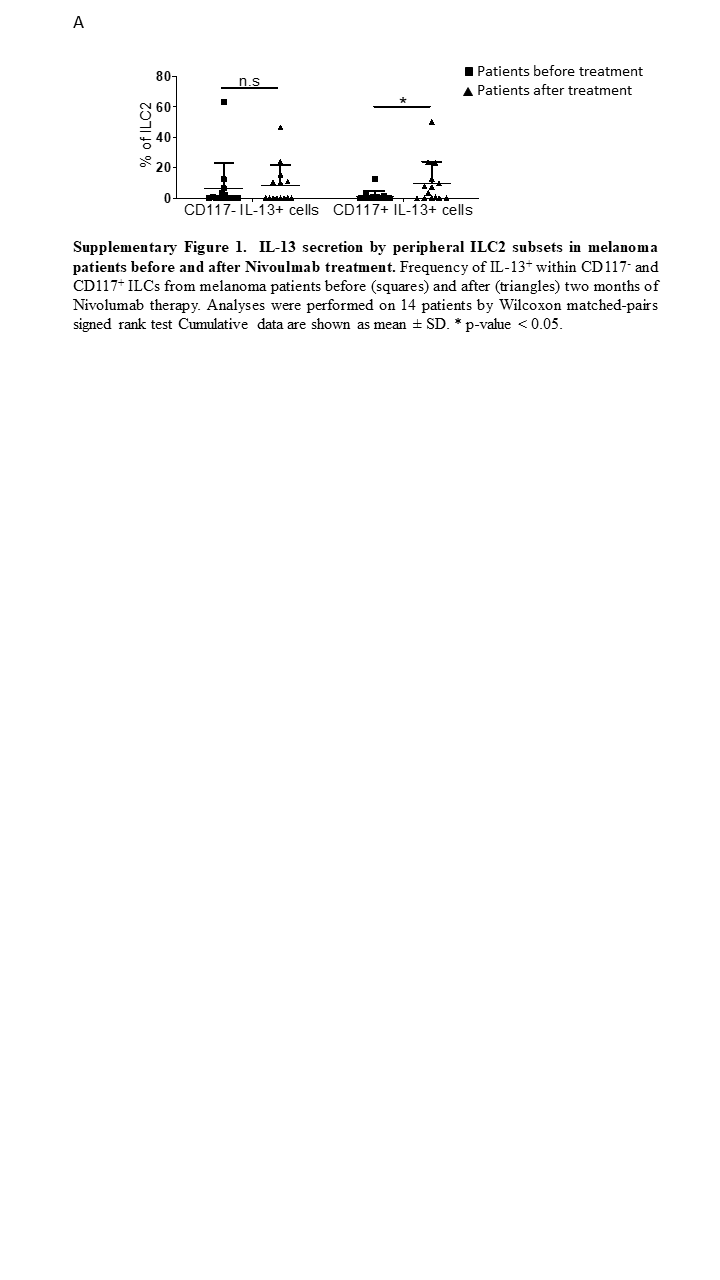

Supplement: Supplementary file 1 [file Image_1.tif]

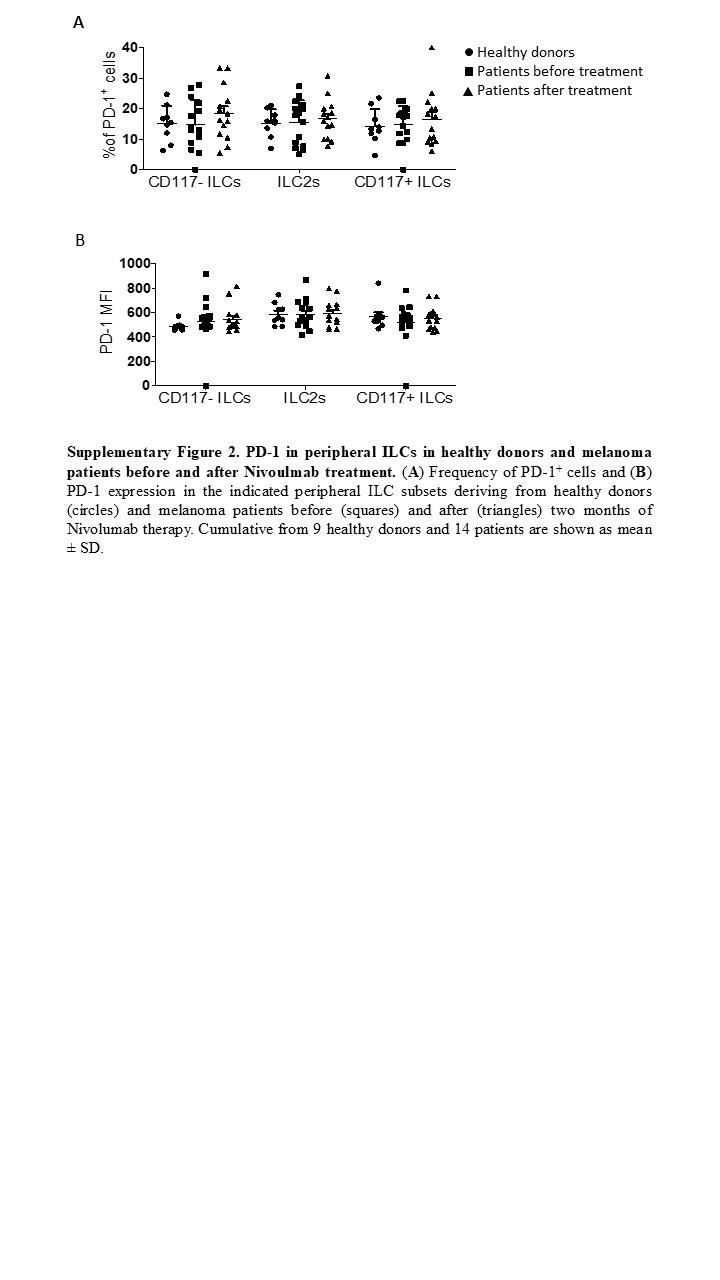

Supplement: Supplementary file 2 [file Image_2.tif]

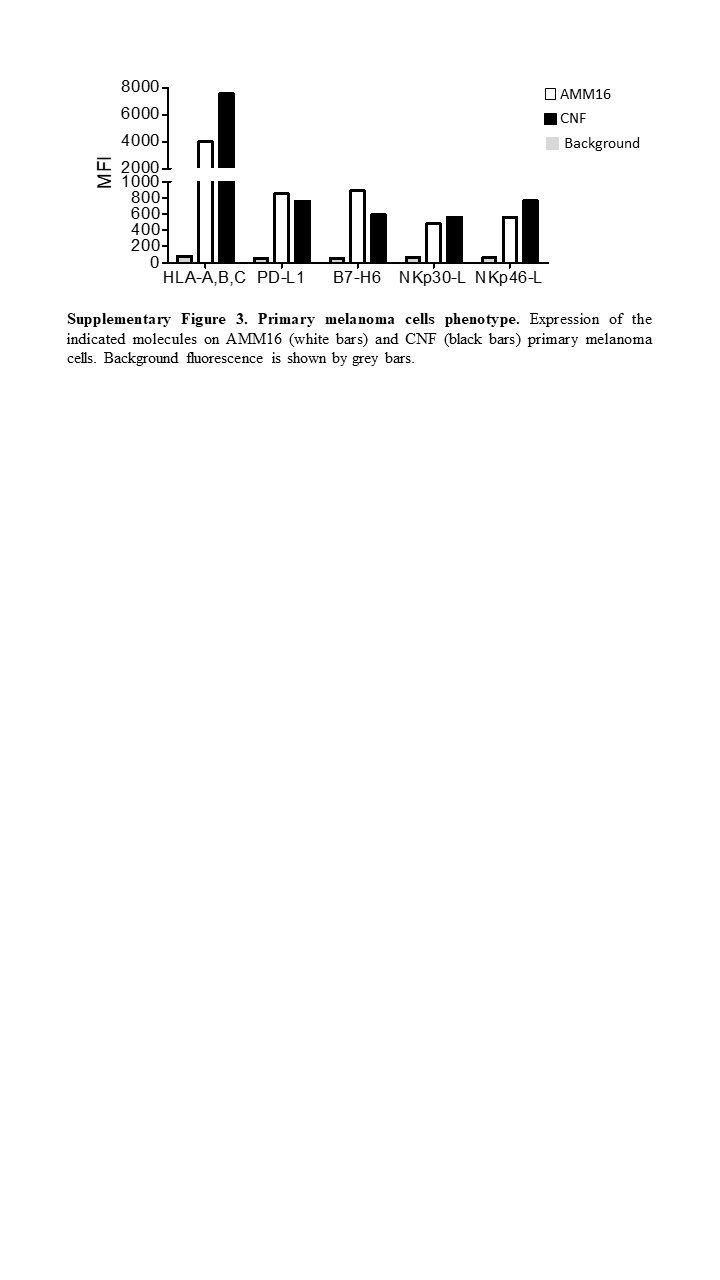

Supplement: Supplementary file 3 [file Image_3.tif]

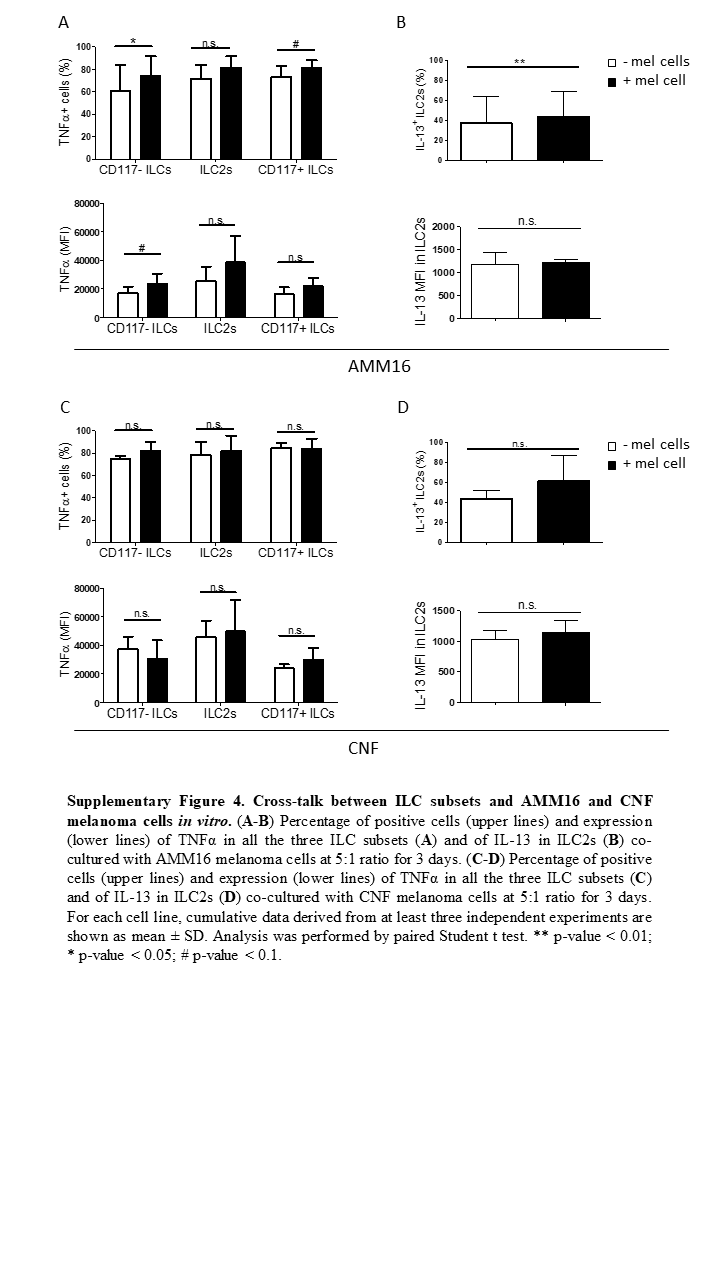

Supplement: Supplementary file 4 [file Image_4.tif]
