# Supplementary material for: Altered Frequencies and Functions of Innate Lymphoid Cells in Melanoma Patients Are Modulated by Immune Checkpoints Inhibitors
Source: Front Immunol. 2022 Jan 31;13:811131. doi: 10.3389/fimmu.2022.811131 (PMC8841353; doi:10.3389/fimmu.2022.811131)
Supplement: Supplementary file 5 [file Table_1.docx]

Supplementary Table 1. Characteristic of Nivolumab- and Ipilimumab-treated stage IV melanoma patients’ cohorts.

| **Variables** | **Nivolumab N (%)** | **Ipilimumab n (%)** |
| --- | --- | --- |
| Number of patients | 24 | 8 |
| Median age | 70.0 (24-85) | 69.0 (37-86) |
| **Sex** |  |  |
| Male | 14 (58.3) | 4 (50.0) |
| Female | 10 (41.7) | 4 (50.0) |
| **Subclass** |  |  |
| M1a | 5 (20.8) | 2 (25.0) |
| M1b | 5 (20.8) | 3 (37.5) |
| M1c | 14 (58.3) | 3 (37.5) |
| M1d | 3 (12.5) | 2 (25.0) |
| **LDH level** |  |  |
| Elevated (>480) | 3 (12.5) | 0 (0.0) |
| Normal | 15 (62.5) | 7 (87.5) |
| Not Available | 6 (25.0) | 1 (12.5) |
| **Previous Therapy** | 18 (75.0) | 4 (50.0) |
| **Subsequent Therapy** | 9 (37.5) | 6 (75.0) |
| **BRAF Status** |  |  |
| Wild Type | 18 (75.0) | 6 |
| Mutated | 6 (25.0) | 2 |
