# Supplementary material for: Altered Frequencies and Functions of Innate Lymphoid Cells in Melanoma Patients Are Modulated by Immune Checkpoints Inhibitors
Source: Front Immunol. 2022 Jan 31;13:811131. doi: 10.3389/fimmu.2022.811131 (PMC8841353; doi:10.3389/fimmu.2022.811131)
Supplement: Supplementary file 6 [file Table_2.docx]

Supplementary Table 2: Antibodies used for cytofluorimetry analysis of PBMCs and cell cocultures as well as for ILCs sorting.

| Marker | Conjuction | Clone/Class |
| --- | --- | --- |
| CD1a | AF488 | HI149/IgG1, k (Biolegend) |
| CD3 | AF488 | HIT3a/IgG2a, k (Biolegend) |
| CD3 | AF700 | UCHT1/IgG1, k (Biolegend) |
| CD4 | FITC | RPA-T4/IgG1, k (Biolegend) |
| CD5 | FITC | UCHT2/IgG1, k (Biolegend) |
| CD11c | AF488 | 3.9/IgG1, k (Biolegend) |
| CD14 | AF488 | HCD14/IgG1, k (Biolegend) |
| CD16 | AF488 | 3G8/IgG1, k (Biolegend) |
| CD19 | FITC | HIB19/IgG1, k (Biolegend) |
| CD34 | FITC | 581/IgG1, k (Biolegend) |
| CD123 | FITC | 6H6/IgG1, k (Biolegend) |
| CD303 (BDCA-2) | AF488 | 201A/IgG2a, k (Biolegend) |
| FceRIa | FITC | AER-37 (CRA-1) /IgG2b, k (Biolegend) |
| TCR α/β | AF488 | IP26/IgG1, k (Biolegend) |
| TCR γ/δ | FITC | B1/IgG1, k (Biolegend) |
| CD127 | PE-Cy7 | A019D5/IgG1, k (Biolegend) |
| CD294 (CRTH2) | PE-CF594 | BM16/IgG2a, k (BD Bioscience) |
| CD294 (CRTH2) | BV421 | BM16/IgG2a, k (BD Bioscience) |
| CD117 (c-kit) | PC5.5 | 104D2D1/IgG1 (Beckman Coulter) |
| CD3 | AF700 | OKT3/IgG2a, k (Biolegend) |
| CD94 | APC-Vio770 | REA113/IgG1 (Miltenyi Biotec) |
| CD56 | BV605 | HCD56/IgG1, k (Biolegend) |
| CD56 | AF700 | HCD56/IgG1, k (Biolegend) |
| CD335 (NKp46) | BV650 | 9E2/IgG1, k (Biolegend) |
| CD336 (NKp44) | PE | p44-8/IgG1, k (Biolegend) |
| CD336 (NKp44) | BV711 | p44-8/IgG1, k (BD Biosciences) |
| CD45RO | BV510 | UCHL1/IgG2a, k (Biolegend) |
| CD279 (PD-1) | AF700 | EH12.2H7/IgG1, k (Biolegend) |
| KLRG1 | Super Bright 702 | 13F12F2/IgG2a, k (eBioscience) |
| CCR10 | BV421 | 1B5/ IgG2a, k (BD Biosciences) |
| CD200R | BV421 | OX-108/IgG1, k (BD Biosciences) |
| NKp80 | APC | 5D12/IgG1, k (Biolegend) |
| CD45 | APC/Fire 750 | 2D1/IgG1, k (Biolegend) |
| EOMES | eFluor 610 | WD1928/IgG1, k (eBioscience) |
| T-bet | BV605 | 4B10/IgG1, k (Biolegend) |
| GATA3 | PE | TWAJ/IgG2b, k (eBioscience) |
| RORγt | AF647 | Q21-559/IgG2b, k (BD Biosciences) |
| TNFα | BV421 | MAb11/IgG1, k (Biolegend) |
| IFNγ | BV510 | 4S.BS/IgG1, k (Biolegend) |
| IL-13 | APC | JES10-5A2/IgG1, k (Biolegend) |
| IL-17a | AF700 | BL168/IgG1, k (Biolegend) |
| IL-22 | PE | 142928/IgG1 (R&D Systems) |
| TNFα | PE-Cy7 | MAb11/IgG1, k (Biolegend) |
| IL-9 | PE-CF594 | MH9A3/IgG1, k (BD Biosciences) |
| IL-5 | BV421 | TRFK5/IgG1, k (Biolegend) |
| IL-17f | BV650 | O33-782/IgG1, k (BD Biosciences) |
| Perforin | AF488 | dG9/IgG2b, k (eBioscience) |
| CD3 | Biotin | OKT3/IgG2a, k (Biolegend) |
| CD4 | Biotin | OKT4/IgG2b, k (Biolegend) |
| CD14 | Biotin | 63D3/IgG1, k (Biolegend) |
| CD16 | Biotin | 3G8/IgG1, k (Biolegend) |
| CD19 | Biotin | HIB19/IgG1, k (Biolegend) |
